# SAEROF: an ensemble approach for large-scale drug-disease association prediction by incorporating rotation forest and sparse autoencoder deep neural network

**DOI:** 10.1038/s41598-020-61616-9

**Published:** 2020-03-18

**Authors:** Han-Jing Jiang, Yu-An Huang, Zhu-Hong You

**Affiliations:** 1 Xinjiang Technical Institute of Physics and Chemistry, Chinese Academy of Science, Urumqi, 830011 China; 2 University of Chinese Academy of Sciences, Beijing, 100049 China; 3 Xinjiang Laboratory of Minority Speech and Language Information Processing, Urumqi, China; 4 Department of Computing, Hong Kong Polytechnic University, Hung Hom, Hong Kong

**Keywords:** Predictive medicine, Predictive medicine, Molecular medicine, Molecular medicine

## Abstract

Drug-disease association is an important piece of information which participates in all stages of drug repositioning. Although the number of drug-disease associations identified by high-throughput technologies is increasing, the experimental methods are time consuming and expensive. As supplement to them, many computational methods have been developed for an accurate *in silico* prediction for new drug-disease associations. In this work, we present a novel computational model combining sparse auto-encoder and rotation forest (SAEROF) to predict drug-disease association. Gaussian interaction profile kernel similarity, drug structure similarity and disease semantic similarity were extracted for exploring the association among drugs and diseases. On this basis, a rotation forest classifier based on sparse auto-encoder is proposed to predict the association between drugs and diseases. In order to evaluate the performance of the proposed model, we used it to implement 10-fold cross validation on two golden standard datasets, Fdataset and Cdataset. As a result, the proposed model achieved AUCs (Area Under the ROC Curve) of Fdataset and Cdataset are 0.9092 and 0.9323, respectively. For performance evaluation, we compared SAEROF with the state-of-the-art support vector machine (SVM) classifier and some existing computational models. Three human diseases (Obesity, Stomach Neoplasms and Lung Neoplasms) were explored in case studies. As a result, more than half of the top 20 drugs predicted were successfully confirmed by the Comparative Toxicogenomics Database(CTD database). This model is a feasible and effective method to predict drug-disease correlation, and its performance is significantly improved compared with existing methods.

## Introduction

The average cost of a successful new drug is estimated at more than $1 billion and the process takes nearly a decade. However, drug repositioning can find some new drug efficacy in both marketed and unlisted compounds, thereby reducing the cycle and cost of drug development. Drug repositioning, also known as new use of old drugs, refers to the process of expanding indications and discovering new targets through further research for drugs that have been on the market. Drug-disease association is an important theoretical basis for drug repositioning. Therefore, the prediction of new drug-disease association has attracted more and more researchers’ attention. In addition to the experimental methods, computational methods to discover new drug-disease associations can lead to further cost savings.

Some researchers have published computational models of drug repositioning based on deep learning techniques. For example, Lu *et al*. used regularized nuclear classifiers to construct drug and disease predictions^1^. Liang *et al*. used a Laplacian regularization algorithm for sparse subspaces to construct a drug repositioning prediction model: LRSSL2^2^. The method incorporates information such as medicinal chemistry information and drug targets. To solve this problem, Wu *et al*. proposed a semi-supervised graph cutting algorithm to find the optimal graph cutting to identify potential drug-disease associations, which is called SSGC^3^.

In the computation framework of most computational methods for predicting drug-disease associations, two modules of feature extraction and classification are normally constructed separately. Effective feature extraction methods could help to improve the prediction accuracy^4^. The similarity between drugs/disease used to be constructed as they are considered to be important to describe their correlation with regards to pattern of drug-disease associations. The first consideration is how to express the features of a particular drug or disease. Therefore, based on the consideration of multiple features, different feature extraction methods are proposed. For example, when DR2DI describing the similarity of the disease, the information content on the disease Medical Subject Headings (MeSH) descriptors and their corresponding Directed Acyclic Graphs (DAGs) are used^5^. In addition to the commonly used machine learning methods to extract features, sparse auto-encoders have recently received attention. For example, Deng *et al*. applied sparse auto-encoder to the study of speech emotion recognition^6^. Su *et al*. used training neural networks to capture the internal structure of the human body^7^. In recent years, with the development of auto-encoder and other types of deep learning technology, some feature extraction methods based on deep learning are gaining more and more research attention. Feature dimension reduction can effectively extract useful features. Using auto-encoder to map the raw features into a low-dimensional space in which the relations of drug and disease can be more effectively measured. In our model, we proposed a feature extraction method combining sparse auto-encoder and PCA to learn the feature representation of drugs and diseases. Sparse auto-encoder is a variant of based auto-encoder, which integrates sparse penalty term into conventional auto-encoder.

In this study, we propose a computational model that combing a sparse auto-encoder with the rotation forest. With a comprehensive consideration of multiple features, we use a combination method to obtain the combined features. A feature extraction module based on sparse autoencoder and Principal Component Analysis (PCA) is established, and the combined features are learned into the final feature representation by sparse auto-encoder. Considering that the ensemble classifier normally yield more stable prediction results than single classifier, we adopt rotation forest to deal with the extracted features from sparse auto-encoder for final prediction. The results yield from rotation forest describe the probability scores of each drug-disease pair to be interactive. Those drug-disease pairs with high prediction scores are considered most likely to be associated among all testing samples.

The results of the SAEROF model after 10-fold cross-validation on Fdataset and Cdataset were compared with the two most advanced drug reposition prediction models. The results show that SAEROF model has better performance. In addition, case studies were conducted on three human diseases, including obesity, Stomach Neoplasms, and Lung Neoplasms. Of the top 20 candidates predicted by SAEROF (Obesity 17/20, Stomach Neoplasms 16/20, Lung Neoplasms 16/20), more than 10 were validated in the CTD database^8^.

## Materials and Methods

In this section, the model we proposed is introduced: First, we describe the datasets used. and second, we explain how to use datasets to calculate similarities between drugs and diseases. Last, the results of the cross-validation rotation forest experiment are given.

**Figure 1:**
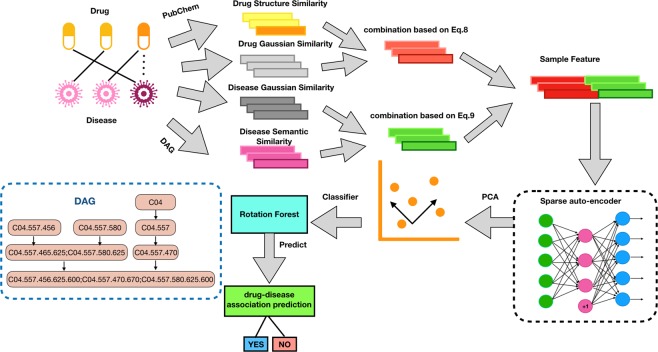
Flowchart of SAEROF model.

Figure 1 is a flow chart of the SAEROF model predicting potential drug-disease associations. First, two kinds of drug similarity and disease similarity were calculated respectively. Then, the feature matrix is obtained by combining drug and disease similarity. Get the final similarity by using the sparse auto-encoder. Finally, a rotation forest classifier is used to predict whether a given drug-disease pair is relevant.

### Dataset

We used the Fdataset and Cdataset collected by Gottlieb *et al*. and Luo *et al*.^9,10^, to predict the drug-disease association. Fdataset contains 593 drugs, 313 diseases and 1933 drug-disease associations. C dataset contains 663 drugs, 409 diseases and 2532 drug-disease associations. (Cdataset is obtained from the previous work (Luo *et al*., 2016^10^), which is generated by combining DNdatasets and Fdataset.)Drug information are extracted from DrugBank and PubChem^11,12^. DrugBank is a database of drugs that contains comprehensive information. PubChem database provides information on the chemical substructure of drugs. The OMIM database provides disease information, which focuses on human genes and diseases^13^. The number of all associations, drugs, and diseases contained in the two datasets is listed in Table 1.

**Table 1:** The data comparison list of the database.

Datasets	Drugs	Diseases	Associations
Cdataset	663	409	2532
Fdataset	593	313	1933

### Similarity for drugs and disease

We here introduce two kinds of drug similarities and two kinds of disease similarities in this section. Drug structure similarity is calculated based on the chemical structure of the drug. Simplified molecular-input line-entry system (SMILE) is a notation that describes the structure of a molecule in a short text string and for a given drug is downloaded from DrugBank^14^. Chemical similarity kits were used to calculate the similarity between the two drugs^15^. Similarities that do not provide prediction information are converted to values close to 0. Next, group drugs based on existing drug-disease relationships. We adjust the similarity by applying the logistic function.

1 $$L(x)=\frac{1}{1+{e}^{(cx+f)}}$$

Such that for $x\in [0,0.3],L(x)\approx 0$,and for $x\in [0.6,1],L(x)\approx 1$. This means that $L(0)$ needs to be as close as possible to zero, so $f$ is $\log (999)$. The value of *c* is determined as −15 by the PRINCE algorithm. The idea of the PRINCE algorithm is: (a) performance comparison of various logistic regression parameters; (b) performance comparison under different iterations; (c) performance comparison of various alpha values.

**Figure 2:**
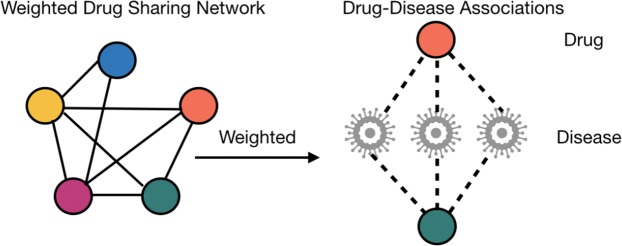
Weighted drug sharing network. The dotted line represents the drug-disease association between, and the shared diseases of drug pairs represent the weight.

Using the above method, drug similarity $D{E}_{r}$ can be obtained. We established a new weighting network for drug sharing (As shown in Fig. 2). Nodes in the drug mapping network, common diseases of drug pairs represent edge weights.

In the SAEROF model, we use ClusterONE^16^ to identify clusters. The definition of cohesion of cluster V is as follows:

2 $$C(V)=\frac{{W}_{in}(V)}{({W}_{in}(V)+{W}_{bound}(V)+P(V))}$$

${W}_{in}(V)$ represent the total weight of the edge in *H*. ${W}_{bound}(V)$ represent the total weight of vertex set and other edges of the group. $P(V)$ represent a penalty term. Assuming that drug ${r}_{i}$ and ${r}_{j}$ belong to the same cluster $V$. Drug structure similarity $DE$ between ${r}_{i}$ and ${r}_{j}$ was defined as:

3 $$DE=(1+C(V))\ast D{E}_{r}$$

It is worth noting that for the structural similarity between drugs, if its value is not less than 1, use 0.99 instead^10^.

Directed acyclic graphs (DAG) can be used to describe semantic similarity of diseases, which can be downloaded from the national liary of medicine’s comprehensive retrieval control vocabulary, medical subject words (MeSH) database^17^. Suppose in $DA{G}_{f(i)}$ of disease $\,b$, the effect of ancestral disease $t$ to disease $b$ is:

4 $$\{\begin{array}{ll}{D}_{f(i)}(b)=1 & if\,b=f(i)\\ {D}_{f(i)}(b)=\,{\max }\{\psi \cdot {D}_{f(i)}({b^{prime}})|{b^{prime}}\in children\,of\,b\} & if\,b\ne f(i)\end{array}$$

Where $\psi$ is the semantic effect parameter, which is related to $b$ and its sub-disease ${b^{prime}}$. In $DA{G}_{f(i)}$, semantic effect of the disease $f(i)$ itself is defined as one. The semantic value $DV(f(i))$ is:

5 $$DV(f(i))=\sum _{s\in {N}_{f(i)}}{D}_{f(i)}(b)$$

The higher the proportion of DAGs sred by the two diseases, the higher the similarity. The semantic similarity score of disease *f*(*i*) and *f*(*j*) is:

6 $$SV(\,f(i),f(\,j))=\frac{{\sum }_{s\in {N}_{f(i)}\cap {N}_{f(j)}}({D}_{f(i)}(s)+{D}_{f(j)}(b))}{DV(\,f(i))+DV(\,f(\,j))}$$

Next, the semantic similarity of disease is improved by using the same measure of drug structure similarity. The similarity was adjusted by analyzing the drug-disease association. Finally, ClusterONE was used to cluster the diseases to obtain the comprehensive similarity $DS$ of the diseases.

Define the adjacency matrix A, where the columns represent the drug and the rows represent the disease. The $i-th$ column vector of the adjacency matrix A is represented by the binary vector $V(g(i))$. Calculate the Gaussian interaction profile kernel of drug $g(i)$ and drug *g*(*j*)^18^:

7 $$GE(g(i),g(j))=\exp (\,-\,{\theta }_{g}{\Vert V(g(i))-V(g(j))\Vert }^{2})$$

8 $${\theta }_{g}={\theta }_{g}^{{\prime} }/\left[\frac{1}{nd}\mathop{\sum }\limits_{u=1}^{nd}{\Vert V(g(u))\Vert }^{2}\right]$$

where Parameter ${\theta }_{g}$ is could adjust the kernel bandwidth and normalize the original parameter $\dot{{\theta }_{g}}$.

Similar to the calculation method of drug similarity, disease Gaussian interaction profile kernel similarity formula is:

9 $$GD(d(i),d(j))=\exp (\,-\,{\theta }_{d}{\Vert V(d(i))-V(d(j))\Vert }^{2})$$

10 $${\partial }_{d}={\partial }_{d}^{{\prime} }/[\frac{1}{md}\mathop{\sum }\limits_{u=1}^{md}{\Vert V(d(u))\Vert }^{2}]$$

where binary vector $V(d(i))$(or $V(d(j)$) represents the association profiles of disease $d(i)$ (or $d(j)$) by observing whether $d(i)$ (or $\,d(j)$) is associated with each of drugs and is equivalent to the $i-th\,$ (or $\,j-th$) row vector of adjacency matrix $A$. Parameter ${\partial }_{d}$ is implemented to adjust the kernel bandwidth and normalize the original parameter ${\partial }_{d}^{{\prime} }$. The value of ${\theta }_{g}^{{\prime} }$ and ${\partial }_{d}^{{\prime} }$ are set to 0.5 for simplicity.

### Feature fusion

In this section, descriptors from multiple data sources are integrated to predict drug-disease associations. The data set contains some unknown drug -disease associations, and the corresponding Gaussian interaction. profile kernel is 0. To solve this problem, we decided to fuse the structural similarity of drugs and the semantic similarity of diseases. This solution can reflect the related characteristics of diseases and drugs from different perspectives.

Drug semantic similarity $DE$ (Eq. 3) was filled in drug Gaussian interaction profile kernel similarity $GE\,$ (Eq. 7) to form drug similarity matrix $SI{M}_{drug}$. The drug similarity $SI{M}_{drug}(g(i),g(j))$ formula for drug $g(i)$ and drug $g(j)$ is as follows:

11 $$SI{M}_{drug}(g(i),g(j))=\{\begin{array}{ll}GE(g(i),g(j)) & if\,g(i)\,and\,g(j)has\,Gaussian\\  & interaction\,profile\,kernel\,similarity\\ DE & otherwise\end{array}$$

For the similarity of diseases, Disease semantic similarity $DS\,$was filled in disease Gaussian interaction profile kernel similarity $GD$ (Eq. 9). The formula is:

12 $$SI{M}_{disease}=\{\begin{array}{ll}GD(d(i),d(j)) & if\,d(i)\,and\,d(j)\,has\,Gaussian\\  & interaction\,profile\,kernel\,similarity\\ DS & otherwise\end{array}$$

### Feature extraction based on SAEROF

**Figure 3:**
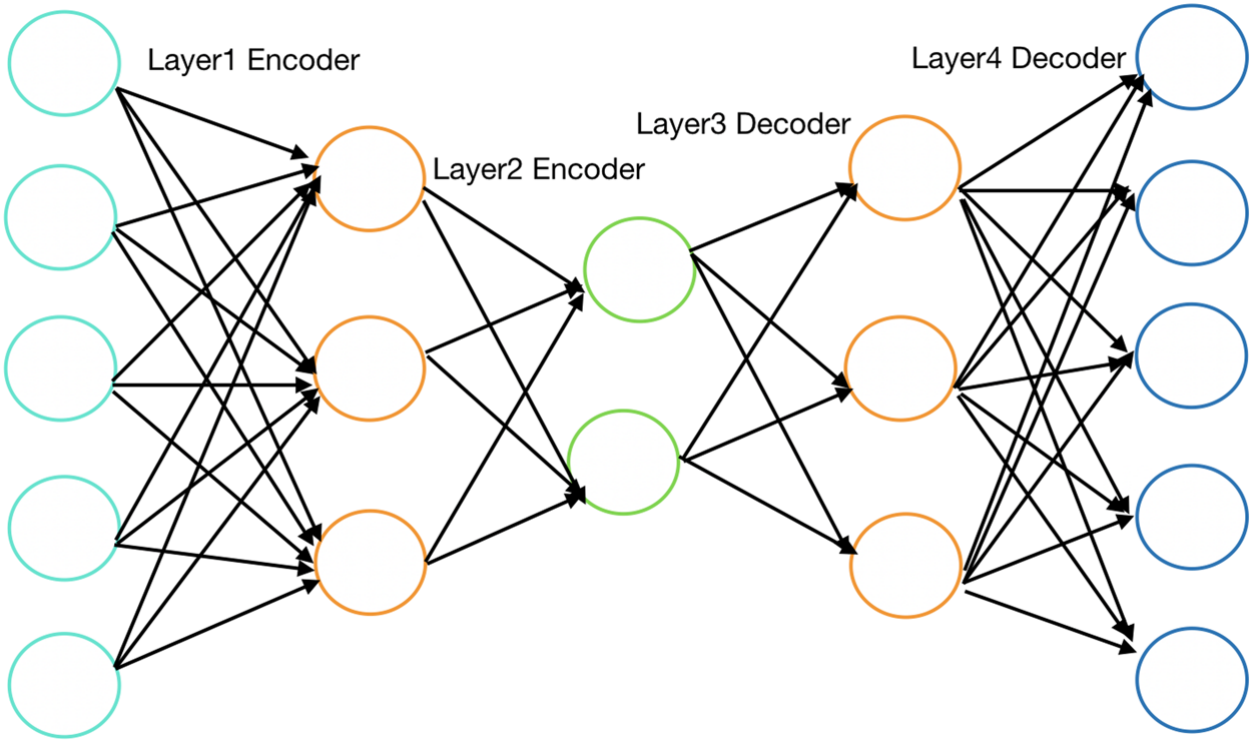
The structure of an auto-encoder.

In recent years, bioinformatics has paid great attention to the application of deep learning. As an effective learning strategy, deep learning is widely used. As an unsupervised neural network model, the autoencoder can learn the hidden features of the input samples. Its basic structure is shown in Fig. 3. However, autoencoders cannot effectively extract useful features. Aiming at this problem, a sparse autoencoder (SAE) is proposed, which introduces a sparse penalty term to learn relatively sparse features.

SAE is a three-layered symmetric neural network. Select $\sigma (x)=1/(1+{e}^{-x})\,$as the activation function of the network. Encoder function,

13 $$h=\sigma ({W}_{encoder}x(i)+{b}_{encoder})$$

The input layer $x$ is mapped to the hidden layer $h$. The decoder function is:

14 $$y=\sigma ({W}_{decoder}h+{b}_{encoder})$$

where *W* represents the connection parameter between the two layers, $b$ is an offset.

Add sparsity penalty to the target function of the auto-encoder to obtain valid features. Suppose ${a}_{j}(x)$ denotes the activation of hidden unit $t$. The average activation amount of hidden unit $t$ is:

15 $$\widehat{{\rho }_{t}}=\frac{1}{n}\mathop{\sum }\limits_{i=1}^{n}[{a}_{t}(x(i))]$$

The sparse term is added to the objective function that penalizes $\widehat{{\rho }_{t}}$ if it deviates significantly from $\rho$. The penalty term is expressed as:

16 $${P}_{penalty}=\mathop{\sum }\limits_{t=1}^{{S}_{2}}KL(\rho \Vert \widehat{{\rho }_{t}})$$

${S}_{2}$ is the number of neurons in the hidden layer. $\rho$ is a sparsity parameter, usually a small value close zero. There is a weight attached to the penalty, which is 10e-7. Kullback-leibler ($KL(\rho ||\widehat{{\rho }_{t}})$) is the relative entropy between two Bernoulli random variables with a mean value of $\rho$ and a mean value of $\hat{\rho }$ ^19^. Relative entropy is a standard measure of the difference between two distributions.

17 $$KL(\rho \Vert \widehat{{\rho }_{t}})=\rho log\frac{\rho }{\widehat{{\rho }_{t}}}+(1-\rho )log\frac{1-\rho }{1-\widehat{{\rho }_{t}}}$$

This penalty function possesses the property that $KL(\rho ||\widehat{{\rho }_{t}})=0$ if $\widehat{{\rho }_{t}}=\rho$. Otherwise, it increases monotonically as $\widehat{{\rho }_{t}}$ diverges from $\rho$, which acts as the sparsity constraint.

The cost function with sparse penalty term added is defined as:

18 $${C}_{sparse}(W,b)=C(W,b)+\gamma \mathop{\sum }\limits_{t=1}^{{S}_{2}}KL(\rho ||\hat{\rho })$$

$C(W,b)$ is the cost function of the neural network. $\gamma$ is the weight of the sparse penalty. As shown in formula 15, the cost function be solved by minimizing *W* and *b*. This can be calculated through the backpropagation algorithm, where the random gradient descent method is used for training. The parameters W and b of each iteration are updated as follows:

19 $${W}_{it}(l)={W}_{it}(l)-\sigma \frac{\partial }{\partial {W}_{it}(l)}{C}_{sparse}(W,b)$$

20 $${b}_{i}(l)={b}_{i}(l)-\sigma \frac{\partial }{\partial {b}_{i}(l)}{C}_{sparse}(W,b)$$

where ${\boldsymbol{\sigma }}\,$is represent the learning rate. The average activation degree is calculated through the forward traversal of all training examples to obtain the sparse error. To optimize the hyperparameters in our models^20^, we keep trying by setting the dimension from 10 to 200. As a result, we found that the performance actually robust to the setting when the dimension is higher than 50^21^. Specially, the performance reaches its highest within the interval of [95,105]. Therefore, the dimension of the hidden layer was optimized as 100.The output layer of Fdataset is 100 dimensions and the input layer is 906 dimensions. The output layer of Cdataset is 100 dimensions and the input layer is 1072 dimensions. We used a single layer sparse automatic encoder. To reduce the computational cost of the classifier, we used the bottleneck hidden layer as the output, which is 100 dimensional. The learning rate is adaptively changed during the optimization by the adadelta algorithm.

Dimensionality reduction is a kind of data set preprocessing technology, which is usually used before the data is applied in other algorithms. It can remove some redundant information and noise of the data, making data more simply and efficiently, so as to improve the data processing speed and save a lot of time and cost. Dimension reduction has also become a widely used data preprocessing method. Principal Component Analysis (PCA) is the most widely used data dimension reduction algorithm. The main idea of PCA is to map n-dimensional features to k-dimensional features, which are brand new orthogonal features and also known as principal components. They are k-dimensional features reconstructed on the basis of the original n-dimensional features. The essence of PCA algorithm is to find some projection directions, so that the variance of the data in these projection directions is the largest, and these projection directions are orthogonal to each other. Here, we reduced the 100-dimensional features obtained by SAE to 84 dimensions through PCA to obtain the final eigenvector.

Ensemble learning complete learning tasks by building and combining multiple machine learning models. Since ensemble learning algorithms are more accurate than single classifiers, they have received more and more attention in recent years. Rotation forest (RF) is a popular ensemble classifier proposed by Rodriguez *et al*.^22^. which has been widely used in various fields. First, RF randomly divides samples into different subsets. Local principal component analysis (PCA) is then used to rotate each subset to increase diversity. Input the rotated subset into different decision trees. The final result of the classification is produced by voting on all the decision trees. Due to the introduction of randomness, RF can prevent overfitting, resist noise and be insensitive to abnormal outliers. Therefore, in this work, we chose the rotation forest as a classifier to process the learned features. We optimize parameters through a grid search, and the parameters of rotation forest, K and n_classifiers are set as 200 and 139, respectively. The ensemble classifier is composed of several weak classifiers, and the subtree selects the feature subset with fewer dimensions. The subtree training is simple as the same as the way to train a decision tree. Its time cost complexity is O(n*|D|*log(|D|)), where |D| is the feature dimension.

## Results and discussion

### Evaluation Criteria

We evaluated the performance of SAEROF by 10-fold cross validation. The evaluation criteria used include precision (Prec.), recall, F1-score and accuracy (Acc.). The calculation formula is defined as:

21 $$Prec.=\frac{TP}{TP+FP}$$

22 $$Recall.=\frac{TP}{TP+FN}$$

23 $$F1-score=\frac{2PR}{P+R}$$

24 $$Acc.=\frac{TP+TN}{TP+TN+FP+FN}$$

TP is defined as a positive sample, which is actually a positive sample. TN is defined as a negative sample, and in fact is a negative sample. FP stands for positive sample, but actually negative sample. FN is defined as a negative sample, but it’s actually a positive sample. In addition, the Receiver Operating Characteristic (ROC) curve and the area under the curve (AUC) that can comprehensively reflect the performance of the model are also used in the experiment.

### Evaluate prediction performance

We chose to use a 10-fold cross-validation method to evaluate the ability of the SAEROF model to predict drug-disease associations. On the Fdataset and Cdataset, all data sets were randomly divided into 10 equal parts. Choose one group at a time as the test set and the other nine as the training set. Finally, the mean and standard deviation of the results of ten experiments were calculated.

**Figure 4:**
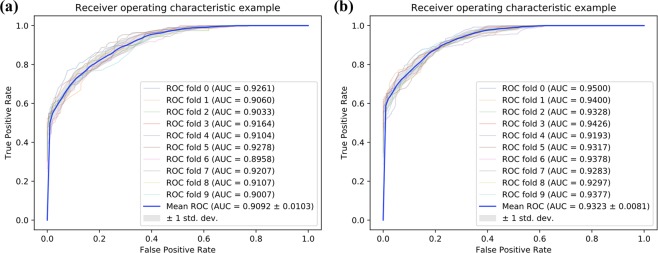
Comparison of ROC curves on Fdataset and Cdataset. (**a**) Is the ROC curve of 10-fold cross validation on the Fdataset. (**b**) s is the ROC curve of 10-fold cross validation on the Cdataset.

Tables 2, 3 and Fig. 4 list the experimental results of SAEROF model on Fdataset and Cdataset. On the Fdataset, the results were as follows: accuracy is 81.17% ± 1.47%, precision is 83.41% ± 1.90%, recall is 77.91% ± 3.35%, f1-score is 80.51% ± 1.75% and mean AUC is 0.9092 ± 0.0103. On the Cdataset, the results were as follows: accuracy is 83.47% ± 1.59%, precision is 85.83% ± 1.17%, recall is 80.21% ± 3.67%, f1-score is 82.87% ± 1.98% and mean AUC is 0.9323 ± 0.0081.

**Table 2:** 10-fold cross-validation results performed by SAEROF on Fdataset.

Test set	Acc. (%)	Pre. (%)	Recall. (%)	F1-score. (%)
0	83.76	83.25	84.54	83.89
1	79.90	82.95	75.26	78.92
2	79.90	79.90	79.90	79.90
3	79.53	80.32	78.24	79.27
4	79.79	84.43	73.06	78.33
5	82.90	84.70	80.31	82.45
6	81.09	86.14	74.09	79.67
7	82.38	83.78	80.31	82.01
8	82.38	85.31	78.24	81.62
9	80.05	83.33	75.13	79.02
Average	**81.17** ± **1.47**	**83.41** ± **1.90**	**77.91** ± **3.35**	**80.51** ± **1.75**

**Table 3:** 10-fold cross-validation results performed by SAEROF on Cdataset.

Test set	Acc. (%)	Pre. (%)	Recall. (%)	F1-score. (%)
0	86.42	87.45	85.04	86.23
1	84.45	84.58	84.25	84.42
2	82.81	85.78	78.66	82.06
3	84.78	84.38	85.38	84.87
4	82.61	86.67	77.08	81.59
5	83.40	84.49	81.82	83.13
6	81.23	85.27	75.49	80.08
7	82.81	86.73	77.47	81.84
8	81.23	85.27	75.49	80.08
9	84.98	87.66	81.42	84.43
Average	**83.47** ± **1.59**	**85.83** ± **1.17**	**80.21** ± **3.67**	**82.87** ± **1.98**

The high accuracy of the SAEROF model stems from the feature extraction method and the choice of classifiers. Combined with sparse auto-encoder, relatively sparse features can be extracted. The ensemble strategy and random tree rotation strategy make the rotation forest classifier have better classification ability.

In order to evaluate the SAEROF model from multiple perspectives, we compared the results with those of two state-of-the-art models, DrugNet and HGBI^23,24^. For all methods we used a ten-fold cross validation. Experiment results (As show in Table 4) show that the AUC of SAEROF is obviously superior to the other two. The AUC values of DrugNet model on Fdataset and Cdataset are 0.778 and 0.804, respectively. The AUC values of the HGBI model on Fdataset and Cdataset are 0.829 and 0.858, respectively. In addition, the AUC values of the SAEROF model on the two data sets are higher than the DrugNet model, respectively. 0.1312 and 0.128, which are 0.0802 and 0.074 higher than the DrugNet model, respectively. The comparison results show that the SAEROF model is significantly better than the other two models. Unlike these two models, the use of sparse autoencoders can learn sparse features and combine with rotation forest classification to obtain more meaningful prediction results.

**Table 4:** AUC Results of cross validation experiments.

Method	Fdataset	Cdatase
DrugNet	0.778(0.001)	0.804(0.001)
HGBI	0.829(0.012)	0.858(0.014)
**SAEROF**	**0.9092**(**0.010)**	**0.932**(**0.008)**

### Comparison among different classifier

**Figure 5:**
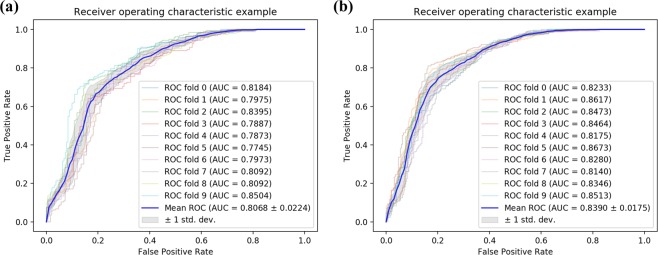
Comparison of ROC curves of SVM classifier in Fdataset and Cdataset. (**a**) Is the ROC curve of 10-fold cross validation on the Fdataset. **(b**) s is the ROC curve of 10-fold cross validation on the Cdataset.

In this section, in order to evaluate the effectiveness of the proposed feature extraction method combined with the rotation forest classifier, an attempt is made to replace the rotation forest classifier with SVM classifier^25^. Tables 5, 6 and Fig. 5 summarize the results of the SVM classifier 10-fold cross-validation on dataset. On Fdataset, the indicators of SVM classifier are: accuracy 74.06% **± **1.83%, precision 71.12% **± **1.83%, recall 81.12% **± **3.62%, f1-score 75.74% **± **1.94% and mean AUC is 0.8068 ± 0.0224. On Cdataset, the indicators of SVM classifier are: accuracy 76.92% **± **1.99%, precision 74.25% **± **2.05%, recall 82.46% **± **2.26%, f1-score 78.13% **± **1.86% and mean AUC is 0.8390 ± 0.0175. It can be seen from the results that the results of the rotation forest classifier are significantly better than the SVM classifier. Due to the idea of ensemble learning and the rotation strategy of the random tree, the rotation forest classifier has better performance than the SVM classifier when using the same feature descriptor.

**Table 5:** 10-fold cross validation used in Fdataset with SVM classifier.

Test set	Acc. (%)	Pre. (%)	Recall. (%)	F1-score. (%)
0	75.52	71.06	86.08	77.86
1	74.23	70.61	82.99	76.30
2	76.55	75.12	79.38	77.19
3	70.73	69.42	74.09	71.68
4	74.35	70.26	84.46	76.71
5	71.50	67.97	81.35	74.06
6	72.28	70.48	76.68	73.45
7	75.39	71.49	84.46	77.43
8	74.35	72.38	78.76	75.43
9	75.65	72.40	82.90	77.29
**Average**	**74.06** ± **1.83**	**71.12** ± **1.83**	**81.12** ± **3.62**	**75.74** ± **1.94**
**SAEROF**	**81.61** ± **1.35**	**78.55** ± **1.78**	**87.07** ± **2.27**	**82.56** ± **1.28**

**Table 6:** 10-fold cross validation used in Cdataset with SVM classifier.

Test set	Acc. (%)	Pre. (%)	Recall. (%)	F1-score. (%)
0	74.61	72.08	80.31	75.98
1	79.13	75.00	87.40	80.73
2	77.87	75.09	83.40	79.03
3	77.47	75.09	82.21	78.49
4	75.69	74.07	79.05	76.48
5	80.63	78.60	84.19	81.30
6	75.69	73.21	81.03	76.92
7	75.10	71.67	83.00	76.92
8	74.51	71.83	80.63	75.98
9	78.46	75.90	83.40	79.47
**Average**	**76.92** ± **1.99**	**74.25** ± **2.05**	**82.46** ± **2.26**	**78.13** ± **1.86**
**SAEROF**	**83.35** ± **1.49**	**81.71** ± **1.60**	**85.98** ± **2.35**	**83.77** ± **1.50**

### Case studies

We implemented the case studies on Fdataset and Cdataset, respectively. Case studies on Obesity and Stomach Neoplasms were carried out on Fdataset, and case studies on Lung Neoplasms were carried out on Cdataset. Specifically, in the experiment, we used Fdataset and Cdataste to train the model. It is important to note that when predicting the drug associated with a disease, all associations between a particular disease and the drug should be removed from the data set. We used the CTD database to validate the top 20 drugs predicted by SAEROF. The World Health Organization has defined obesity as diseases that pose a threat to human health, manifested by excessive accumulation of fat. Obesity is major threats to many chronic diseases, including diabetes, cardiovascular disease and even cancer. We selected obesity as the first case study and used SAEROF to predict related drug. As shown in Table 7, after comparing prediction results with the CTD dataset, 17 of the top 20 predicted drugs were confirmed.

**Table 7:** The top-20 drugs predicted to be associated with Obesity.

Index	Drug Name	Evidence	Index	Drug Name	Evidence
1	Topiramate	Confirmed	11	Benzphetamine	Confirmed
2	Sibutramine	N.A.	12	Methotrexate	Confirmed
3	Phenylpropanolamine	Confirmed	13	Prednisone	Confirmed
4	Phentermine	Confirmed	14	Mitoxantrone	Confirmed
5	Phendimetrzaine	N.A.	15	Scopolamine	Confirmed
6	Orlistat	Confirmed	16	Imipramine	Confirmed
7	Methamphetamine	Confirmed	17	Dexamethasone	Confirmed
8	Diethylpropion	Confirmed	18	Azathioprine	N.A.
9	Cimetidine	Confirmed	19	Diazepam	Confirmed
10	Bupropion	Confirmed	20	Clonazepam	Confirmed

Stomach Neoplasms are common digestive disorders that are both benign and malignant. We selected this disease as a case study to validate the predictive power of SAEROF. Table 8 lists the 20 drugs that SAEROF predicts are highly associated with Stomach Neoplasms. Comparison with CTD database shows that 16 of the top-20 drugs predicted by Stomach Neoplasms can be identified.

**Table 8:** The top 20 drugs predicted to be associated with Stomach Neoplasms.

Index	Drug Name	Evidence	Index	Drug Name	Confirmed
1	Terazosin	Confirmed	11	Diethylpropion	N.A.
2	Tacrolimus	Confirmed	12	Beclomethasone	Confirmed
3	Spironolactone	Confirmed	13	Baclofen	Confirmed
4	Meloxicam	Confirmed	14	Prazosin	Confirmed
5	Hyoscyamine	N.A.	15	Metoclopramide	N.A.
6	Glatiramer acetate	N.A.	16	Methotrexate	Confirmed
7	Famotidine	Confirmed	17	Memantine	Confirmed
8	Escitalopram	Confirmed	18	Thalidomide	Confirmed
9	Carbamazepine	Confirmed	19	Ibuprofen	Confirmed
10	Phenobarbital	Confirmed	20	Gliclazide	Confirmed

The incidence and mortality of Lung Neoplasms have increased significantly in recent decades. We chose lung tumors on Cdataset as case studies to verify SAEROF’s predictive power. As shown in Table 9, comparing the predicted results with the CTD data set, 16 of the top 20 predicted drugs proved to be associated with Lung Neoplasms.

**Table 9:** The top 20 drugs predicted to be associated with obesity Lung Neoplasms.

Index	Drug Name	Evidence	Index	Drug Name	Confirmed
1	Pyridoxine	Confirmed	11	Gemcitabine	Confirmed
2	Etoposide	Confirmed	12	Alprostadil	Confirmed
3	Felbamate	Confirmed	13	Fluocinolone acetonide	Confirmed
4	Levocabastine	N.A.	14	Doxazosin	Confirmed
5	L-Alanine	Confirmed	15	Etidronic acid	Confirmed
6	Cetirizine	Confirmed	16	Medrysone	N.A.
7	Lamotrigine	Confirmed	17	Spermine	Confirmed
8	Auranofin	Confirmed	18	Donepezil	Confirmed
9	Alimemazine	N.A.	19	Decitabine	N.A.
10	Loratadine	Confirmed	20	Piroxicam	Confirmed

Case studies of obesity, Stomach Neoplasms and Lung Neoplasms have shown that SAEROF performs well in predicting the most promising drugs.

## Conclusion

In order to further accelerate the process of drug repositioning, effective methods for predicting drug-disease association are urgently needed. Our model opens up new perspectives for predicting drug-disease associations. In the feature extraction process, three kinds of descriptor, Gaussian interaction profile kernel, drug structure similarity and disease semantic similarity are extracted from the drug-disease association pair. The representative features are extracted using sparse auto-encoder. Finally, the rotation forest classifier is used for sample classification.

Experiments have shown that the SAEROF model is suitable for large-scale prediction of drug-disease associations, and the results of case studies on obesity, Stomach Neoplasms, and Lung Neoplasms confirm this view. In order to further improve the accuracy of the prediction model, protein information and disease gene information can be integrated in the future.

## Data Availability

The datasets that we collected in this work is freely available on https://github.com/HanJingJiang/SAEROF.
